# The Nature and Treatment of Chorea

**Published:** 1911-03

**Authors:** Carey F. Coombs

**Affiliations:** Assistant-Physician, Bristol General Hospital


### THE NATURE AND TREATMENT OF CHOREA.

BY

CAREY F. COOMBS, M.D., M.R.C.P. Lond.,
*Assistant-Physician, Bristol General Hospital.*

CHOREA is a tiresome malady even when it is not worse than tiresome; it keeps the child away from school, interferes with his play, and exposes the impotence of the doctor. This is, I believe, because we do not allow ourselves to appreciate the nature of the disease, and the inevitable limitations of treatment. The remarks which follow express ideas as to its nature formed from a clinical study of over 200 cases, and *post-mortem* examination of four other cases.

### THE NATURE OF CHOREA.

(*a*) **Its rheumatic origin.**—The first point is that Sydenham's chorea is a manifestation of rheumatic infection; no rheumatism, no chorea. This is supported by the following tabulated figures:—

| Where seen. | Total cases of chorea. | Cases with history of articular rheumatism | Cases with carditis or nodules, but no history of articular rheumatism. | Cases developing articular, cardiac, or nodular lesions later (*i.e.* after but not before chorea). | Total cases with some form of rheumatic manifestation before, with, or after chorea. |
|---|---|---|---|---|---|
| St. Mary's Hospital, In-patients. | 70 | 23 = 33 per cent. | 21 = 30 per cent. | Not Investigated. | 44 = 63 per cent. |
| Bristol General and Children's Hospitals, Out-patients. | 157 | 85 = 54 per cent. | 33 = 21 per cent. | 10 = 6.4 per cent. (*i.e.* 10 out of 18 cases followed up.) | 128 = 81 per cent. of whole number; 44 per cent. of whole number after exclusion of cases not followed up. |

That is to say, of a total of 227 cases observed for periods varying from a few weeks up to five years, 172 (or 76 per cent.) either give a rheumatic history or show definite evidence of rheumatic infection. I may add that only definite cases of cardiac disease have been considered as evidence of cardiac rheumatism ; those types of variation from the normal which leave one in doubt as to whether they should be considered evidence of disease or not have not been counted. As to the small percentage not accounted for, it would be strange indeed if a general infection—such as rheumatism in childhood certainly is — did not sometimes manifest itself by nervous phenomena only, considering the high specialisation of the nervous tissues and their consequent sensitiveness to infective poisons. Further, there are patients who forget their rheumatic attacks, so that a history of rheumatism may be denied when it should be acknowledged.

It may be argued, and with perfect truth, that choreiform movements occur in other infections, and that they have to all appearance been provoked by iodoform poisoning. The answer to this is that choreiform movement is not the same thing as "chorea." By the term "chorea" we understand a certain definite nervous disorder, the most striking feature of which is a particular kind of muscular disturbance, from which the disease takes its name. (As will be shown presently, chorea is more than a mere motor disorder.) The difference between this and the choreiform movements which may occur in the course of staphylococcal or streptococcal infection, or in a case of cerebro-spinal meningitis, is that the former is a peculiarly persistent nervous disease, while the latter are but a phase, and usually a brief phase, of some definite generalised infection which only rarely provokes choreiform movement. Careful inquiry will always show that the most persistent cases are really a series of attacks intermitting for short intervals, but never leaving the patient quite normal. I have notes of several children whose lives, for as many as five years, consisted of attacks of chorea once or twice a year, separated by gradual but incomplete convalescences. And this is one of the very close resemblances between chorea and other rheumatic phenomena: the articular pains of rheumatic infection in childhood are always recurring, several times a year, in association with brief periods of fever. Again, if we keep children with apparently quiescent carditis under careful observation for prolonged periods we find frequent bursts of mild pyrexia accompanied by a quickening of the pulse and a temporary or enduring enlargement of the heart. Even the nodules themselves come out in crops, quite numerous in some cases. Progress by repetition of acute attacks may indeed be described as a cardinal feature of rheumatic infection. We find, then, *evidence of the rheumatic nature of Sydenham's chorea in its almost invariable association with other manifestations of rheumatic infection; and in its characteristically "rheumatic" course, a series of active attacks separated by incomplete recoveries.*

(*b*) **Post-mortem Changes in Chorea.**[1]—Granted, then, that rheumatic infection causes chorea, how does it do so? Perhaps the best answer to this is to be found in a consideration of the changes found in the central nervous system after death in fatal cases of chorea. In two of these cases death was due to concurrent carditis, in the other two to bulbar poisoning. I do not propose to give a lengthy account of these changes in the present paper, but merely to mention the salient features of four cases examined by myself. My findings agree in essentials with those of other investigators, though in my cases certain features were lacking which have been remarked upon by some. The special cells of the central nervous system showed marked degenerative changes, contrasting rather conspicuously with an almost normal condition of the connective tissues and blood vessels. These degenerative changes vary from the earliest stage of chromatolysis to absolute destruction of the internal cellular detail. The cortical cells suffer most severely, practically all of them being more or less injured, many of them presenting no recognisable structure. All parts of the cortex exhibit a similar type and

[1] Part of the expense of the investigations mentioned here was defrayed by a grant from the British Medical Association.

degree of alteration; the motor cortex is damaged neither more nor less than the cortex from the temporal, frontal, occipital, orbital, calloso-marginal and other areas. Changes of the same kind, but of a less severe degree, are to be seen in the optic thalamus and the mesencephalic grey matter generally. A certain relatively slight amount of chromatolysis is to be noted in the motor nuclei of the pons and medulla; but the anterior cornual cells of the cervical cord show not the least departure from the normal. The cerebellum was not diseased. In my own cases there have been none of the meningeal changes mentioned by some observers; there have been a few capillary thromboses and occasional peri-capillary exudations of cells, but these are, I believe, evidences of death and not of infection. It may be said here that in two of the cases several hundreds of sections of pia mater were examined, so that it is not likely that any inflammatory foci were present but overlooked. Certain it is that there were none of those microscopical nodules which have been found by various German observers as well as by myself to be characteristic of rheumatic myocarditis, and which I have also found in the pericardium and in the valvular and mural endocardium in rheumatic carditis, and in the synovial membrane from one rheumatic joint—nodules which are similar in structure to the larger subcutaneous nodes so characteristic of rheumatic infection. I have not found micro-organisms in my sections; but I do not insist on this, as the material was not specially chosen for that purpose.

It is this *contrast between a normal state of the meninges and widespread and intense cortical changes* to which attention must first be drawn. It seems to suggest that there may be an intoxication of the brain without any actual invasion of the cranial contents by micro-organisms. In certain cases of chorea, it is true, the clinical picture suggests the presence of some meningitis, and in one or two instances examination of the cerebro-spinal fluid and of the meninges themselves has practically proved that a rheumatic meningitis can occur; but these cases are exceptional to the general rule. The discovery of micro-organisms in the meninges and in the cerebro-spinal fluid in severe or fatal cases of chorea does not prove a special selection of the brain by organisms any more than the discovery of B. typhosus in the cerebro-spinal fluid of enteric patients. Further, there is some clinical support for the intoxication theory of chorea. Miller's[1] very interesting paper on "latent chorea" shows that in a large proportion of all children showing rheumatic manifestations nervous instability can be detected, in the shape of uncertainties of temper, occasional choreic movements, a liability to night terrors, and a peculiarly definite state of general misery. My own cases furnish examples of this. In the case of a girl of fifteen, whom I had seen several times before with attacks of articular rheumatism and progressive carditis, when she came to hospital with so severe an attack that I had to recommend her for admission, she burst out into violent screaming at the top of her voice, the exact counterpart of the behaviour of children with chorea. A Sister at the General Hospital once remarked to me, quite spontaneously, that of all the children admitted to her ward the rheumatic subjects were the most miserable, always fretting and crying about things that other children would ignore. This persistent state of unrest, described very clearly by Miller, is, I am convinced, a most definite feature of the influence of the rheumatic infection upon children, and it seems more likely to be due to persistence of the organisms in some part of the body (*e.g.* the lymphatic tissues or the alimentary tract) and out-pouring of poisons into the blood-stream than to a constant, steady microbic invasion of the brain. Of course, there is a middle theory: chorea may be due in most cases to a general "septicæmic" distribution of organisms in the intracranial tissues, while the cardiac, subcutaneous and articular nodules, as well as the meningitic cases of chorea, may be perhaps due to a "pyæmic" scattering of organisms in groups or small masses throughout the body. This is, however, mere speculation, profitless except in so far as it prompts further research.

The next point of interest to be noted in connection with the pathological data is the fact that *the cortex suffers much more*

[1] Miller, *Lancet*, 1909, ii, 1808.

*than the rest of the central nervous system.* In the cortex a normal cell can scarcely be found ; in the pons and medulla there were as many cells normal as diseased, and none were quite destroyed. The cervical cord was unaffected. This fact bears directly upon the nature of the choreic movements—an important matter, for before making up our minds as to the best way of checking these movements we must have some conception of their mode of origin, as to whether they are due to removal of inhibitory influences or to direct irritation of cortical cells. Anyone who has seen sections of the cortex from fatal cases of chorea will agree that the cells are so universally injured that the disease has in all probability attacked those which exercise functions of high control over the motor cells, as well as the motor cells themselves. It is impossible that in the general destruction any function can have escaped. If this be so, we may take it that the rheumatic toxins act upon the cortex in the same way as do other cerebral intoxicants in accordance with the laws of dissolution. The highest level areas of the cortex go first, so that loss of control is the first functional result, the motor cells themselves being attacked later. And clinical facts are in accord with this ; the earlier stages of chorea are marked by loss of power to control emotion, and by movements which are really voluntary effort run wild. The child, reaching out for a cup, projects the hand too far and too fast ; the cup is upset, yet the movement was volitional in conception. In later stages of the disease, and especially in severe cases, movements that are quite involuntary dominate the picture, until the unfortunate patient, writhing and tossing ceaselessly, appears to have solved the problem of perpetual motion. These involuntary movements point not only to a lack of control by damaged high level centres, but also to a diffuse, persistent irritation of the motor cells, which are being destroyed by the poison of rheumatic infection, and they are accompanied by lack of voluntary power due to the same cause. In some cases and at some stages, indeed, paresis of voluntary effort is the chief motor symptom ; perhaps this represents a minor degree of cellular injury. Finally, the disease may end, as Dr. J. A. Nixon noted in one of the two cases which he kindly allowed me to investigate pathologically, with symptoms suggestive of paralysis of the respiratory centre. That this interpretation is probably correct is supported by the discovery of very definite chromatolytic changes in the motor cells of the medullary nuclei in the case alluded to.

It has been suggested that the chromatolysis noted in cortical cells after chorea is due to fatigue, the result of continuous and prolonged muscular exertion. That this is unlikely is shown by the fact that all parts of the cortex are damaged alike, and by the normal condition of the anterior cornual cells of the cervical cord.

(*c*) **Symptomatology.**—Another point of great importance in its bearing on the treatment of chorea is emphasised and explained by the catholicity of distribution of the cortical changes. I refer to the fact that *chorea is not a mere motor disorder, an outbreak of movements, but an organic disease showing its effects in disturbance of various functions of the central nervous system.* It follows that treatment must include a great deal more than mere suppression of movements : it must accomplish restoration of healthy structure and function to all the injured neurones. A brief summary of the symptomatology of chorea, as it has appeared in my own cases, will bring out the truth of this statement. The symptoms are, for the sake of convenience, grouped under the headings which I use in examining and recording cases of nervous disease.

(1) *Psychical.* Delirium with visual hallucinations is seen in bad cases. The commoner symptoms are lack of emotional control, as shown by irritability, easily provoked laughter and violent sudden outbursts of crying about " nothing," and delay in answering questions. Whether reaction-time has ever been measured in choreics I do not know, but there can be no doubt as to the general fact that cerebration is slow. The power of mental concentration is also diminished.

(2) *Cranial.*—Langmead[1] has alluded to certain pupillary phenomena, notably hippus, which I have seen in some cases.

[1] Langmead, *Lancet*, 1908, 1, 154.

Transient squint is also present in a few instances, and one child complained of diplopia. Corneal and pharyngeal anæsthesia will be referred to below. In one case a period of aphonia occurred.

(3) *Motor.*—The affections of movement fall under three headings, as has already been said—loss of inhibition, involuntary irritative movements, and paresis.

(4) *Sensory.*—The sensory phenomena of chorea have been investigated by various physicians, notably Purves Stewart[1] and certain French observers.[2] My own examination of this point has been restricted to a few cases, not quite representative of chorea in general, because it proved useless to expect accurate replies from young children, so that my cases include a large proportion of older patients. Of the twenty-one who were examined for sensory changes, there were five in whom some definite diminution of the tactile sense was noted. In three of these there was a more or less definite hemianæsthesia of the trunk and limbs. In one of the other two cases there was relative anæsthesia below the knees, a bilateral "stocking area"; and in the other the sense of touch and pain was so definitely lessened in the left arm that the patient complained of it himself spontaneously.. In one other case there seemed to be a diffuse diminution of the pain sense, which could not be delimited within any particular area. In five cases also there seemed to be unilateral or bilateral diminution of corneal sensation; this was, however, so indefinite that it cannot be insisted upon. I did not find any clear instance of pharyngeal anæsthesia, but Miller has recorded examples.[3] The only relation between alteration of cutaneous sensibility and the other features of chorea that was noted in my cases was that it seemed associated with the severer grades of the disease. There was no clear relation between the distribution of the sensory and that of the motor phenomena.

(5) *Co-ordination* is, of course, affected on the motor side by the liability to overdo the volitional movements fostered by

[1] Stewart, *Edinb. M. J.*, 1899, N.S., v, 257.

[2] Babonneix, *Ann. de Mal. d. Enf.*, 1908, xi, 816. [3] Miller, *Loc. cit.*

the lack of higher control. Whether perversions of the muscular sense occur, and play any part in causing incoördination, I do not know. In one case a distinct intention tremor was found in movements of the arms. Miller[1] has remarked upon the occasional occurrence of nystagmus.

(6) *Reflexes.* Certain of these were investigated in fifty-five cases. In forty-three of these the knee-jerks were abnormally easy to elicit ; in one case they were almost absent ; in one other they were difficult to get, while in the remainder they were normal. In four cases the exaggeration was more marked on one side ; in three of these the movements were also more marked on that side. In sixteen cases one or both knee-jerks were of the " hung-up " type described by Dr. William Gordon, of Exeter ; of the eight cases in which one knee-jerk was more definitely "hung" than the other, four were noted as showing more movement on that side, while in two cases there was paresis on that side ; in one other case the movements were symmetrical in intensity, and in the remaining one the hanging-up of the knee-jerk was more definite on the side on which both movements and paresis were less distinct. In one case the knee-jerks, though hard to elicit, were of the " hung-up " order when they did appear.

The arm-jerks (triceps and biceps) were tested in twenty cases ; in fourteen of these they were unusually easy to elicit ; in six of these the abnormal readiness was more marked on one side, and in several a " hung-up " response to stimulation of the triceps insertion was noted. Ankle clonus is noted in one of the fifty-five cases. As for the plantar response, it was flexor on both sides in forty-nine cases, extensor on both sides in one case, extensor on one side in four cases, and indeterminate in one case. In three of the cases with unilateral extensor response it occurred on the side of more movement in three cases and on that of less movement in one case. All those cases showing extensor response were severe examples of the disease.

(7) *Trophic.*—There are no symptoms falling under this heading.

[1] Miller, *Loc. cit.*

(8) *Sphincters.*—The incontinence which is seen in some cases of chorea, both of urine and of fæces, is due to sudden violent movements of the trunk, raising the intra-abdominal and intra-pelvic tension and " forcing " the sphincters. In extremely severe cases there may be paralytic incontinence.

This catalogue of symptoms will show clearly that chorea is much more than a motor disorder. Further, the occasional occurrence of a plantar extensor response on the one hand, and of alterations of sensation similar to those noted in hysteria on the other, in the same disease, is an interesting commentary on the validity of the distinction between " functional " and " organic " nervous disease. The plantar extensor response is usually considered proof positive of organic disease implicating the upper motor neurone ; while " stocking " areas of altered sensibility and hemianæsthesia are often spoken of as strong evidence of the functional nature of a nervous disorder. The lesson that chorea has to teach in this connection is that there is no such thing as a " functional " nervous disorder ; perverted function must needs be accompanied by alteration of structure, and the term " functional " is merely a confession of the inadequacy of the methods available for the demonstration of finer anatomical changes. Chorea is therefore passing from the category of " functional " to that of " organic " disease, as one research after another establishes the existence of definite changes in the cortical cells, detectable by the evolution of more delicate microscopic methods. Here mention may be made of three cases of hysterical contracture of the hand. All occurred in girls of ten to fourteen, all of them suffering from rheumatic carditis, and one of them recovering from chorea. The number is small, but definite hysteria is not very common in girls of that age, so that the association may be more than a coincidence.

(*d*) **Prognosis.**—One last point remains for consideration, before summarising the data as to the nature of chorea upon which the principles of treatment should be based. When we remember the extraordinary persistence of chorea, and the severity of the symptoms in some cases, it is remarkable that recovery should be so complete as it is. It is most difficult, and indeed almost impossible, to discover the slightest trace of enduring cerebral injury in persons who have suffered and recovered from chorea, even in its most protracted forms. It is true that Forssner's[1] interesting researches show that chorea is likely to leave a tendency to general invalidity, even in patients who have escaped the chief danger of rheumatic infection, that of permanent cardiac disease; but the fact remains that the brain, though it has been subjected to the prolonged action of rheumatic toxins, recovers completely, in the great majority of cases, once the cause has been removed. This may be compared with the ultimate effect of the same toxæmia on another specialised parenchyma, that of the myocardium, whose cells in like manner regain health as the patients pass out of the years of adolescence into adult life, out of the period of active rheumatic infection into that of obsolescence. The inference is that these toxins are not intensive so much as extensive in their effects; and that rheumatic infection is of chief importance because of its persistence, and not on account of its virulence.

Concentrating these remarks on the nature of chorea into one sentence, we may say that *it is an organic disease implicating the cells of the cortex cerebri, and those of the mid-brain and ponto-medullary nuclei in a less severe degree; that it is due to the action of rheumatic toxins upon these cells; that all parts of the cortex suffer equally, and therefore the disease should not be regarded as one of motion only; and that, in spite of its extraordinary persistence, due to repetition of active phases, the general tendency is towards recovery.*

### TREATMENT OF CHOREA.

Be it noted, then, that in treating chorea, we are confronted by a condition with which we are not unfamiliar, disease of neurones caused by the action of poisons upon them; a condition such as is exemplified in alcoholic and other forms of multiple toxic neuritis. As in such conditions,

[1] Forssner, *Jahrb. f. Kinderheilk*, vol. lxxi. No. 1.

so in chorea, the principles of treatment are clear ; it is the method of applying them that is not so definite. First, the cause must be removed ; second, the neurones must be rested and thereby allowed to return to health ; third, their recovery will be hastened by improvement of the general health ; and lastly, certain symptoms may call for special treatment.

(*a*) *Removal of the Cause.*—Once the rheumatic origin of chorea is acknowledged, one great step towards the proper treatment has been made. Chorea is to be treated like other manifestations of active rheumatic infection, by rest in bed and administration of salicylates, while there is any evidence of activity. By " evidences of activity " are meant such phenomena as pyrexia (however slight), arthritic pains, the appearance of subcutaneous nodes, tachycardia, loss of flesh, increase in the gravity of the choreic movements and of the mental state, increasing dilatation of the heart, and persistent or increasing anæmia. The action of salicylates is a matter of controversy ; my own belief is based upon a general impression and not upon any figures, for it is quite impossible to measure the duration of an attack of chorea in accurate terms. It is not possible, for example, to describe an attack as beginning on October 1st and ending on November 10th ; probably there were symptoms in September which escaped notice, and it is equally certain that no one could allege certainly that there were no choreic movements on November 11th. This being so, it is not possible to assert that the average duration of an attack is (let us say) three weeks under salicylate treatment, and six weeks under other methods. My own feeling about salicylate treatment is that moderate doses given during that stage of chorea in which the lesions are being established will influence the progress of the disorder favourably. In chronic cases, already on their gradual road to recovery, there is no need to aim at " removing the cause " ; the cause, active rheumatic infection, has already removed itself. Neither are we to expect that chorea will be aborted by salicylate treatment ; for if even the salicyl compounds were absolute and perfect specifics for the destruction of the infective agents and the toxins of rheumatism, we must recollect that when treatment begins the toxins have in all probability already entered into indissoluble chemical union with the protoplasm of the cortical cells; and that no specific except time can undo so fast a bond as this. The usefulness of the salicylates lies in their power of preventing some part of the further damage which might otherwise be done by the toxins of rheumatism. My own experience is that huge doses of sodium salicylate do not act more powerfully in chorea than a moderate dose of ten to twenty grains every four hours, varying according to the patient's age. In severer cases the dose should be more frequently given, every hour or two hours, for the first twelve or twenty-four hours. Twenty or thirty grains of bicarbonate of soda should be given with it, and the bowels must be kept open by salines given at fixed intervals. It should be gradually left off when the active period of infection seems to have ceased; and the same remark applies to the rest in bed which is absolutely necessary in the earlier stages. This "rest" is to be mental as well as physical; children should be kept quiet, isolated if possible, and allowed books and toys in small doses only.

(*b*) *Remedial rest for the neurones.*—Recovery actually begins as soon as the active stage ceases, but since the nerve cell is a highly-organised structure, its recovery is very gradual, so that the perversion of its functions persists for some time after the toxins have ceased to act upon it. It follows that no one can certainly determine the point at which the period of active disease ends and that of convalescence begins. It is therefore impossible to lay down any certain law as to the indications for dropping the treatment by salicylates and rest in bed; one must judge each case according to its own merits. Fortunately it does not matter much, so long as one makes a rule of erring on the side of excess in relation to the length of the period during which these are to be used, generally a period of about a fortnight, and parents should be warned that the attack is not likely to pass away *completely* in less than six weeks, and that any undue forcing of convalescence will mean relapse. Even when the child is well enough to be up and about, choreic movements are likely to be in evidence, though slowly decreasing in degree ; and these are to be considered as proof that the cortical neurones are but imperfectly recovered, and therefore in need of yet more rest. This period of rest should include a lazy life of getting up late and going to bed early, with plenty of fresh air, and regular but not wearying exercise. School is, of course, quite out of the question, for months after the movements have ceased. All this is very difficult to secure in the homes of people living in large towns with limited means ; sometimes it can be compassed by sending the child to stay in the country, if there are relatives available who can be depended upon to allow freedom and fresh air without allowing license and fatigue. When this can be done convalescence is often remarkably shortened. I have not found large convalescent homes very suitable for such children ; there is too much going on, and the excitement of unlimited play with other children interferes with the brain-rest that is needed. The smaller homes often provide a good environment. Exercises and games, wisely supervised and sytematically practised, are valuable in restoring precision of movement.

(*c*) *Improvement of the general health.*—To this end two things are chiefly necessary, fresh air and plenty of food. Whenever it is possible, children recovering from chorea should spend the greater part of the daylight hours out of doors ; this is of course conditional on quiet surroundings, and is another strong argument for convalescence in the country. As to food, ordinary meals should be allowed, with at least one pint of milk daily in addition. Tonics are also of value ; the usual mixture of cod liver oil and malt with Parrish's Food is as good as any, though I think its value is enhanced by the addition of two, three, or four drops of Fowler's solution in each dose. Probably the extraordinary veneration shown by the medical profession for the *treatment of chorea by large doses of arsenic* is in part due to the observation of beneficial effects from small doses, and also in part to a vague feeling that arsenic does good in nervous diseases. With an ingenuity worthy of a better cause, many have devised methods of giving large doses of arsenic to choreics in such a way as to avoid toxic effects. All this is so much energy wasted, for even with large doses of arsenic it is not possible to shorten the course of chorea appreciably. My notes include 25 cases, all of whom had at least half a drachm of Fowler's solution per diem for one, two or more weeks ; in some there were toxic symptoms, in others there were not (owing to the manner of administration in mixture with food), but in no case were the miraculous results noted which we have been led to expect. One girl indeed took an ounce of Fowler's solution in ten days, and was so little the better that her parents took her out of the Hospital, and shortly afterwards I saw her appearing in a local newspaper as the heroine of a wonderful cure by certain potent pills for pale people. After all, it is hardly to be expected that the effects of toxins upon cortical cells will be dispelled by the administration of a drug which also has a selective toxic action on neurones.

(*d*) *The treatment of symptoms.*—The chief symptom calling for treatment is movement in excess. Such treatment is not needed in the great majority of cases of chorea, only in those who are being exhausted by constant activity or prevented by it from sleeping and feeding. Various measures may be used to this end ; only two will be mentioned here. The first is *chloretone.* This drug has been recommended for routine use in chorea, but if we accept the view that the movements unless severe do not call for suppression we shall avoid the use of drugs of this type in all but those few cases in which the excess of muscular activity calls for special measures. It has indeed been my experience (in 22 of my cases where its administration is recorded) that small doses in mild cases tend to aggravate the movements, probably by increasing the failure of inhibition, which, as we have seen, is an important factor in such cases, while larger doses produce a general cerebral depression which is out of proportion to the severity of the movements in all but bad cases. It should be given, when the movements are so bad as to call for some sedative, in doses of four or five grains three times a day, one of the doses being given at bedtime. The dose may be cautiously increased to eight grains in children over ten. The only form in which I have given it is in solution in petroleum emulsion. It appears to act as a muscular depressant as well as a general sedative, for a disproportionate degree of muscular weakness in discernible after its use. The ideal plan is to get the patient well under the influence of the drug, and keep him in a sleepy state for several days, varying according to the severity of the case. As the patient gradually emerges from this state the movements will in most cases be found to have lost their severity. Chloretone is discussed here at some length, because of the prominence given to it of late by several writers in relation to chorea.

When every drug, including chloretone, fails, it is sometimes possible to suppress the most violent movements by means of *hot packs.* This measure has the advantage that it does no harm and is finite in its action.

Another symptom which is often troublesome during convalescence is paresis. In some cases massage has been of considerable value in removing this trouble. There does not seem to be any very good reason for this; none the less, it is a method worth using in such cases.

### SUMMARY.

1. Sydenham's chorea is a manifestation of rheumatic infection.

2. It is an organic disease of the brain, attacking all parts of the cortex equally and impartially, but affecting the basal grey matter less severely.

3. Consequently the symptoms are not only motor, but also psychical and sometimes sensory.

4. The motor symptoms are partly due to loss of inhibition, partly to cortical irritation.

5. In chorea the fallacy of the distinction between "functional" and organic nervous disease is exemplified.

6. Chorea can be fatal without the occurrence of meningeal inflammation.

7. This fact, with the phenomena of latent chorea, suggests that chorea may be due to direct intoxication rather than gross infection of the cranial contents.

8. The tendency of chorea is towards restoration of health to the brain.

9. Treatment consists of (*a*) removal of the cause, active rheumatic infection, by rest in bed with administration of salicylates during the active stages ; (*b*) prolonged mental and bodily rest during convalescence ; (*c*) improvement of general health by fresh air, full diet, and tonics ; (*d*) quieting of excessive movement by sedative drugs or packs, and cure of paresis by massage.

10. The full-dose arsenical treatment is useless.

11. Chloretone is only useful in certain cases, and should not be given as a routine measure.

In conclusion, it is once more my pleasant duty to thank my colleagues at the Bristol General Hospital, and especially Dr. Newman Neild, for their kindness in placing their patients at my disposal for observation ; also Dr. J. A. Nixon, Physician to the Bristol Royal Infirmary, for kindly allowing me to make pathological investigations of two fatal cases ; Dr. Reginald Miller, Assistant-Physician to St. Mary's Hospital, London, for forwarding me pathological material ; and Professor Walker Hall (Professor of Pathology in the University of Bristol) for his unfailing helpfulness in matters of pathological technique.

